# Towards an optimized paradigm: generative adversarial networks and 3D modeling in landscape design and generation

**DOI:** 10.1371/journal.pone.0330095

**Published:** 2025-11-14

**Authors:** Ming He

**Affiliations:** Institute of Design, Chongqing Industry Polytechnic University, Chongqing, China; Macau University of Science and Technology, MACAO

## Abstract

Virtual reality (VR) integrates technologies like computer graphics, artificial intelligence, and multi-sensor systems, creating transformative tools for designers and users. This study proposes a novel urban landscape design method using 3D laser scanning combined with frame reorganization and texture mapping. Despite the advancements in VR-based landscape design, existing methods often suffer from inefficiencies in rendering time and suboptimal visual fidelity, limiting their practical application in large-scale urban projects. In the initial phase, we acquire the central pixel point of the images via a meticulous 3D scanning process, thus facilitating a three-dimensional stereo reorganization of urban architectural landscapes. This stage is succeeded by the application of a terahertz wave image segmentation strategy, grounded in the sophisticated utilization of adversarial generative networks and a structured texture mapping procedure. This technique permits the virtual reconstruction of the architectural blueprint, wherein each image layer is systematically traversed, engendering a dynamic representation of the urban landscape. The final step generates realistic urban landscape simulations using integrated 3D laser scanning. To ascertain the efficacy of the proposed methodology, we embarked upon a series of performance assessments across four disparate simulation design scenarios, yielding verifiable outcomes. Our empirical findings demonstrate that the proposed method reduces rendering times by up to 90% compared to traditional tools like SketchUp and 3D Studio Max, while achieving a significant improvement in visual fidelity, as evidenced by standard image quality metrics. These results attest to the formidable potential of this avant-garde approach within the VR landscape design milieu, significantly diminishing the time imperative while augmenting visual fidelity and fortifying automatic display proficiencies. By virtue of its robust analytical underpinnings and innovative approach, this research furnishes a substantial theoretical scaffolding for the evolving discourse in landscape space design, prompting a reevaluation of conventional methodologies while propelling the field towards a more efficient and visually immersive future.

## 1 Introduction

In contemporary years, there has been an unprecedented acceleration in economic and technological advancement, giving rise to detrimental impacts on the environment concomitant with rapid economic proliferation. Current societal inclinations underscore the pivotal role of fostering aesthetically harmonious urban landscapes as a vehicle to enrich urban environments [1]. Consequently, the meticulous design of urban landscapes has ascended to the forefront of urban development endeavors. Urban landscape design serves as a crucible for the efficacious and judicious planning of vegetation, architectural elements, floral arrangements, and man-made infrastructures, facilitated through the optimization of 3D imaging techniques. Despite these advancements, existing methods often suffer from inefficiencies in rendering time and suboptimal visual fidelity, limiting their practical application in large-scale urban projects. For instance, studies by Borkowski & Nowakowski [2] and Hajirasouli et al. [3] have highlighted the challenges in achieving real-time rendering and high-quality visuals with traditional tools like SketchUp and 3D Studio Max, which struggle with the computational demands of complex urban scenes. This arena presents a significant conduit for the exhibition of city images, augmenting the quality of urban life and bolstering the operational efficiency of urban infrastructural components. The advent of digitalization, propelled by strides in computer technology, has ushered in an era of heightened standardization and scientific rigor in landscape construction, spanning preliminary geological verification to simulated environment settings. However, the reliance on manual drafting and rigid design paradigms has historically amplified workloads and hindered adaptability, as noted by Matthews et al. [4]. Before digital tools, designers used manual drafting, which required advanced skills and significantly increased workloads.

The digital epoch, catalyzed by the widespread accessibility of personal computers, has revolutionized the design landscape, mitigated the workload while enhanced work quality. It eliminates rigid, non-modifiable design paradigms, enabling fluid communication between designers and clients, which improves efficiency and quality. VR technology addresses communication gaps by creating seamless interfaces for stakeholder-designer interaction. Recent studies, such as those by Zhou et al. [5] and Souza & Fabricio [6], have demonstrated the potential of VR in facilitating preemptive evaluations of design harmony, yet they also point to the need for more efficient and visually accurate simulation methods to fully realize its benefits in large-scale projects. Within this framework, cities envisaging futuristic plans can leverage VR to simulate post-completion scenarios, facilitating pre-emptive evaluations of harmony with existing architectural ethos, thereby preventing post-implementation disruptions.

Furthermore, VR technology empowers designers to conceive simulated environments in computer applications, fostering pragmatic solutions that enhance the caliber of design outputs. This evolving technoscientific landscape has transmuted the creative dynamics in design, intertwining artistic sensibilities with scientific innovation.

The confluence of science, technology, and artistic nuances has emerged as a cardinal principle in modern landscape planning and design. Contemporary landscape design necessitates a multifaceted approach encompassing visual aesthetics, ecological sustainability, and harmonious integration with surrounding environments. However, prevalent landscape design systems fall short in meeting the escalating demands of modern landscape planning. For example, researchers used GIS to build a 3D landscape sculpture planning system aimed at improving urban landscape design [7]. However, the visualization effect of the constructed virtual landscape model is not satisfactory enough.

The literature has proposed various strategies to enhance 3D VR design methodologies for urban landscapes [8]. These strategies include intelligent particle swarm optimization-based virtual design technologies and template matching processing. However, existing approaches consistently face limitations in real-time performance [9], adaptive following, and large-scale design intelligence.Specifically, Do al. [10] and Janovský [11] found that current optimization techniques struggle with the computational demands of high-fidelity rendering in complex urban scenes, underscoring the need for more efficient algorithms.

Addressing these identified gaps, this study introduces a novel urban landscape design strategy predicated on the frame reorganization and texture mapping facilitated by 3D laser scanning. Preliminary experimental results corroborate the timeliness and precision of the proposed method, underscoring its potential applicability in landscape optimization endeavors, thus presenting substantial value in engineering applications.

## 2. State of the art

VR technology constitutes an interdisciplinary domain that integrates advanced technologies encompassing computer hardware and software technologies, computer graphics, sensor technologies, ergonomics, human-computer interaction theories, and multimedia technologies [12]. Its deployment spans a diverse array of sectors including but not limited to aerospace, healthcare, industrial arenas, commerce, architecture, education, and entertainment, exhibiting substantial promise for future advancements [13].

The advent of VR and holography has instigated a paradigm shift in how professionals across different domains interact with digital information. This review collates perspectives from four distinct studies encompassing aerospace, medical, 3D displays, and architectural sectors, delineating the advancements and implications of VR and holography technologies therein. Tadeja et al. (2020) [14] introduce AeroVR, an immersive visualization system tailored for aerospace design and digital twinning in VR. The study delineates how AeroVR facilitates a nuanced understanding of aerospace designs, thereby expediting the design process and enhancing precision. The concept of digital twinning, mirrored in VR, presents a powerful tool for real-time monitoring and analysis. Javaid and Haleem (2020) [15] delve into the burgeoning application of VR in the medical realm. Their exposition underlines the instrumental role of VR in medical training, surgical simulation, and patient education, elucidating the potential for significant enhancements in clinical outcomes and educational efficacy. Blinder et al. (2022) [16] provide a comprehensive overview of the state-of-the-art in computer generated holography for 3D display. The article elucidates the technological advancements in rendering realistic 3D visuals, highlighting the profound implications for interactive displays and consumer electronics. Bashabsheh et al. (2019) [17] and Tadeja, S. K. et al. [18] explore the integration of VR technology in architectural education, particularly in building constructions. The study underscores the pivotal role of VR in fostering a hands-on, interactive learning environment, thereby bridging the gap between theoretical knowledge and practical application. The reviewed articles collectively accentuate the transformative potential of VR and holography across diverse fields. The integration of these technologies not only augments visualization and interaction but also holds promise for significantly enhancing educational and operational efficacy. However, challenges such as hardware limitations, user adaptability, and cost factors warrant further exploration.

Historically rooted in the United States, the VR sector has witnessed significant contributions from this region, positioning it as a global frontrunner in VR developments [19]. For instance, the University of Los Angeles pioneered the creation of a robust virtual city management system, fostering a more methodical approach to urban simulation research [20]. Leveraging this system, urban designers have facilitated the redesign of architectural complexes and assisted urban managers in making data-driven decisions to enhance urban aesthetics. Furthermore, the utilization of VR technology in geological explorations, as demonstrated in the earthquake analysis in Mexico undertaken by the University of California, underscores its potential in geological investigations [21].

European nations have also embarked on leveraging VR technologies, with Germany initiating VR-based urban construction designs as early as 1991, yielding remarkable outcomes [22]. Other nations including the Netherlands, Sweden, and France have undertaken comprehensive exploratory and analytic endeavors in VR technology, with firms such as the Dutch Act 3D utilizing Quest3D application for architectural and landscape simulations [23].

Contrastingly, the trajectory of VR technology development in other regions exhibits a more gradual progress, characterized by a discernible gap compared to developments in nations like the United States. However, collaborative initiatives between technology corporations and institutes such as the Tsinghua Design Institute signal a promising direction with substantial strides in 3D visualization and digital city portrayals [24]. Several enterprises are spearheading innovations in 3D animation, simulation imagery, and VR product development, earmarking their presence in significant projects such as earthquake simulations and World Expo exhibitions [25]. Additionally, some entities are focusing on scenic virtual technologies and simulation reality systems, enhancing the hardware and software landscape in computer graphics [26]. Noteworthy is the fostering of global collaborations and exchanges, facilitating technological advancements in sectors such as aerospace through products like the 3D Virtual Air Crew Training System [27].

Despite these advancements, there remains a critical need to further explore VR technology. This is especially evident in virtual landscape design, where substantive and scientific research is still lacking. The forthcoming endeavors in VR technology development necessitate a focused approach on comprehensive and scientific research, delineating the path for future investigations and developments in the sector.

## 3. Methodology

### 3.1 Urban landscape 3D laser scanning image acquisition and pre-processing

#### 3.1. 1 VR 3D laser scanning of urban landscape.

To enhance the rationality of urban landscape design, we employ 3D VR technology for urban landscape image acquisition [7]. This enables the creation of visual spatial data and road-related details for urban landscapes. The distribution of informational features for acquiring scene state information in urban landscapes is outlined as follows.


W(i,j)=w(i,j)+d(i,j) (1)

Where w(i,j) representing the parallax function for integer-level 3D laser scanning of buildings and road structures, while d(i,j) signifies the parallax function for 3D VR imaging of urban landscapes. We employ 3D laser scanning technology for rendering VR imagery of the urban landscape. The process begins with edge contour detection in the urban landscape’s VR imagery using the surface grid detection method. Subsequently, the laser-scanned urban landscape images are segmented within the laser transmission space. Finally, we perform 3D statistical analysis on the laser-scanned urban landscape images through the Monte Carlo (MC) method. The result is a dynamic laser virtual image derived from the 3D laser scanning of the urban landscape.

For example, in a practical scenario, Equation (1) is used to calculate the parallax between the laser-scanned point cloud and the VR image for a specific building. By inputting the coordinates (i,j) of a point on the building’s façade, w(i,j) provides the depth information from the laser scan, while d(i,j) gives the corresponding depth in the VR model. This allows us to align the real-world scan with the virtual design, ensuring accurate placement of design elements like greenery or lighting.


S(i)=Y(i)n(i)+A(1−n(i)) (2)

**Fig 1:**
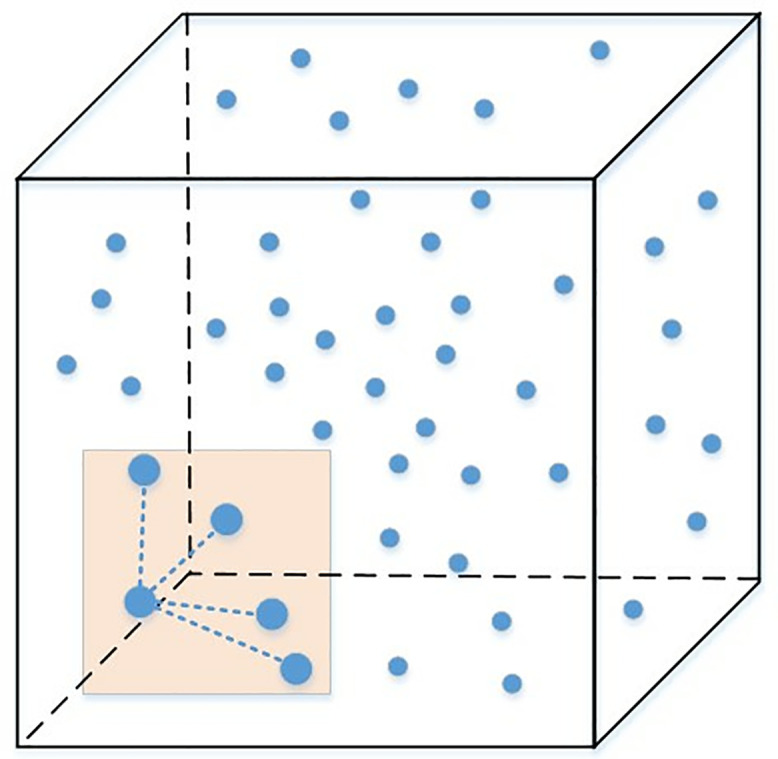
Distribution of 3D laser scanning area of the urban landscape.

Where *A* represents the pixel intensity of the dynamic imaging of 3D laser scanning in the *i*-direction within the urban landscape; n(i) denotes the statistical attribute associated with the dynamic imaging of 3D laser scanning in the urban landscape; Y(i)n(i) stands for the adaptive distribution function in the context of 3D laser scanning of urban landscapes. For the edge contour detection in the VR imaging of urban landscapes, the surface grid detection approach is implemented. The 3D laser scanning procedure is depicted in Fig 1.

To illustrate, consider a scenario where we are scanning a park area with trees and benches. Equation (2) helps in determining the pixel intensity *A* along a specific scanning direction, which is crucial for distinguishing between different materials, such as the rough texture of tree bark versus the smooth surface of a bench. This differentiation aids in accurate texture mapping during the virtual reconstruction phase.

The pixel values within the 3D laser-scanned image are mapped to a 2D coordinate system. The mean of the attenuation coefficient I sub-samples of laser light intensity is employed as an approximate solution for the pixel sequence Z, serving the purpose of VR design for the urban landscape.


$$\begin{array}{c} \overset{-}{i_{N}}=\frac{1}{N}\sum {\mathrm{\backslash nolimits}}_{x=1}^{N}\;i_{x} \end{array}$$ (3)

Where: $i_{\text{1}}\text{,}i_{\text{2}}\text{,}i_{\text{3}}\text{,}$ and so forth, up to $i_{N}$ represent the 3D resampling feature sequence of the urban landscape from image I; N signifies the time interval for laser point scanning; Q(a) corresponds to the limited variance of the continuous function ‘a’ across the region R = [0, 1]. For edge pixel points N points $i_{\text{1}}\text{,}i_{\text{2}}\text{,}i_{\text{3}}\text{,}\cdots \text{,}i_{N}\in R$, a 3D laser-scanned image of the urban landscape consisting of several thread blocks is obtained. The edge contour pixel point distribution satisfies the following equation.


$$U\left(\left|\overset{-}{i_{N}}-Z\right|<\frac{\lambda_{\chi }\sigma }{\sqrt{T}}\right)\approx \frac{2}{\sqrt{2\pi }}\int_{0}^{\lambda^{\chi }}\;e^{-\frac{1}{2n^{2}}}dn=1-\chi$$ (4)

Then, the dynamic reconfiguration of the 3D structure of the urban landscape design is performed based on the central pixel points of the laser 3D scanning imagery. A terahertz wave image segmentation technique based on adversarial generative networks is used for feature extraction to obtain more realistic generated images.

The generator architecture used in this paper consists of multiple residual blocks, and each residual block contains a set of parallel convolutions within it to increase the network depth. Also, the 4-way parallel design with different convolution sizes enables the residual blocks to extract richer image features in the same size of the receptive field. The resolving power of the imaging system is limited by the sensor and the working wavelength, and often fails to reproduce the edge and detail information of the image. The adversarial generative network-based approach can rely on the learning of a small number of samples to achieve computational imaging. The ultimate goal of the approach in this study is to train a generating function G that outputs the corresponding material segmentation image based on the input image $S_{t}$. The parameters of the generator feed-forward convolutional neural network are denoted as $G_{\theta G}$, where $\theta G=\left\{M_{\text{1}:L};h_{\text{1}:L}\right\}$ denotes the weight and bias of the L-th layer of the network. Optimization learning is performed by a specific $l_{G}$ loss function to obtain the corresponding $S_{t}^{F}$. θG is then optimized by Equation (5) to make the results closer to the true label $S_{t}^{\text{Mask}}.$


$$\begin{array}{c} \hat{\theta }G=arg\;\underset{\theta G}{min}\;\frac{1}{T}\sum {\mathrm{\backslash nolimits}}_{t=1}^{T}\;l_{G}\left(G_{\theta G}\left(S_{t}\right),S_{t}^{\text{map}}\right) \end{array}$$ (5)

This paper proposes a specialized loss function ($l_{\text{BCE}}$) to enhance the generator’s ability to deceive the discriminator and produce precise segmented images. The function connects the deep network features of both components, enabling continuous parameter optimization through adversarial training. As a weighted loss function with multiple parameters, the adversarial network gradient is avoided to disappear too quickly while ensuring the image quality. The discriminator is optimized by Equation (6) so that the discriminator accurately distinguishes the real labels from the generated fake images.


$$\begin{array}{c} \hat{\theta }D=arg\;\underset{\theta D}{min}\;\frac{1}{T}\sum {\mathrm{\backslash nolimits}}_{t=1}^{T}\;l_{BCE}\left(S_{t}^{\text{Mask}},1\right)+l_{BCE}\left(G_{\theta G}\left(S_{t}\right),0\right) \end{array}$$ (6)

Inception contains four 1 × 1 convolution blocks, two 3 × 3 convolution blocks, and one 5 × 5 convolution block [28]. The generators are guaranteed to obtain more size image information in the same perceptual field by different size convolutional kernels to normalize the image; finally, the ReLU function is used for activation.

Traditional adversarial networks use convolutional networks to extract image feature maps, and then use fully connected layers to judge the feature maps to generate the discriminant matrix. The number of fully connected layers directly determines the accuracy of the discriminant matrix. Due to the high computational complexity of the fully connected layers, it requires a large number of computational resources and hardware support, resulting in the network depth cannot be increased indefinitely. This article combines the attention mechanism with convolutional networks. Convolutional network images have a receptive field for determining the position of image information, while the attention mechanism is different. It allows the model to have a better representation of the neighborhood information through a priori position information, by assigning different initial values to the η,μ,ζ vectors through three independent fully connected layers, denoted as $\eta M_{x}^{z}\text{,}\mu W_{x}^{v}\text{,}\zeta W_{x}^{v}$, respectively.


$$\cdots \cdots MultiHead(\eta ,\mu ,\zeta )=Concat\left({head}_{1},{head}_{2},\cdots ,{head}_{b}\right)$$ (7)

$${head}_{x}=Attention\left(\eta M_{x}^{z},\mu M_{x}^{v},\zeta M_{x}^{q}\right)$$ (8)

$$Attention(\eta ,\mu ,\zeta )=Softmax\left(\eta \mu^{N}/\sqrt{D_{b}}\right)q$$ (9)

**Fig 2:**
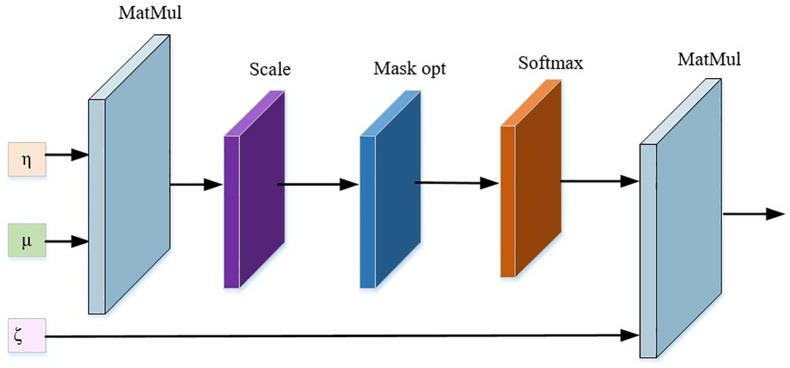
Schematic diagram of the attention mechanism.

Where: η is the query matrix; μ is the target attention content; ζ is the feature value matrix. $D_{b}$ is the scale factor, and using $D_{b}$ prevents the gradient from vanishing after too large dot product is mapped through Softmax. Since the extracted vectors contain multiple sub-image feature blocks, this paper uses Equation (7) and Equation (8) to calculate the image feature vectors that match the marked regions in the feature map. The computational flow of the attention mechanism module in the discriminator consists of 1 multi-headed attention mechanism layer, 1 Multi-Layer Perceptron (MLP) layer, and 2 Linear Normalization (LN) layers connected with the residual information. The outputs of the individual attention mechanisms are superimposed as the real outputs of the module, where the basic principle of the attention mechanism is shown in Fig 2.

The setting of the loss function directly affects whether the generator can update the parameters in the correct direction to generate a more realistic segmented image. In this Section, we further optimize the design based on the loss function in the Super-Resolution Generative Adversarial Network (SRGAN) [29]. The conventional loss function utilizes Mean Square Error (MSE), see Equation (10). However, the MSE-based optimization approach does not perform well on the test set, and the loss of the generator will be the same as the traditional architecture if it is directly derived from the real labels. Therefore, the only way to try to learn more feature information is to increase the depth and width of the network. In this study, we update the generator using the loss function shown in Equation (11) by updating the generator with the feature maps learned within the discriminator, instead of letting the generator learn the label information directly. This gradient update approach ensures that the generator’s parameter updates rely entirely on information derived from the discriminator. As a result, the generator achieves robust generalization performance across diverse test datasets.


$$\begin{array}{c} l_{MSE}=\frac{1}{R^{2}MB}\sum {\mathrm{\backslash nolimits}}_{i=1}^{RM}\;\sum {\mathrm{\backslash nolimits}}_{j=1}^{RB}\;{\left(S_{i,j}^{Mask}-G_{\theta G}{\left(S_{t}\right)}_{i,j}\right)}^{2} \end{array}$$ (10)

$$\begin{array}{c} l_{G\theta }=\frac{1}{M_{x,y}B_{x,y}}\sum {\mathrm{\backslash nolimits}}_{i=1}^{M_{xy}}\;(D_{\theta x,y}\left(S_{i,j}^{Mask\;}\right)-D_{\theta x,y}{\left(G_{\theta }{\left(S_{t}\right)}_{i,j}\right)}^{2}\; \end{array}$$ (11)

Where: $D_{\theta x\text{,}y}$ is the weight and bias parameter θ of the discriminator after x Inception layers and y attention mechanism layers. The discriminator uses the Binary Cross Entropy (BCE) loss function, as shown in Equation (12). The cross-entropy function can effectively make the model maintain the gradient for the binary classification problem. Make the output of the $D_{\theta }\left(S^{\text{Mask}}\right)$ discriminator input real label an all-1 matrix and make the output of the $D_{\theta }\left(G_{\theta G}\left(S_{n}\right)\right)$ discriminator input fake image an all-0 matrix.


$$\begin{array}{c} l_{BCE}=\sum {\mathrm{\backslash nolimits}}_{x=1}^{T}\;-j_{x}lt\left(i_{x}\right) \end{array}$$ (12)

This paper uses the weighted sum of 2 loss functions to update the generator, see Equation (13).


$$\begin{array}{c} l_{G}=Cl_{G\theta }-E\sum {\mathrm{\backslash nolimits}}_{x=1}^{T}\;lt\left(G_{\theta G}\left(S_{t}\right)\right) \end{array}$$ (13)

Where [C,E] is the weighting weight. The generator is updated based on the feature maps learned by the discriminator and whether the fake images generated by the generator can “fool” the discriminator. By learning the feature maps from the discriminator, the generator is more likely to fool the discriminator, thus facilitating model adversarial learning and generating more realistic images.

#### 3.1.2 Texture segmentation of urban landscape images.

The study integrates adaptive feature decomposition for point scanning with dynamic recognition of urban landscape images. Using texture mapping, the laser 3D-scanned image rays are systematically traversed layer by layer. Then, according to the texture parameters indexed by laser ray threads, the texture parameters $\left|\overset{-}{i_{N}}-Z\right|<\frac{\lambda_{\chi }\sigma }{\sqrt{N}}$ of the VR view distribution of the fruitful urban landscape are calculated to be equal to 1- χ. For example, when χ = 0. 01, χ is called the significant level and 1- χ is called the confidence level. In general, when the scale feature quantity χ of the urban landscape VR design satisfies the condition of local convergence, the urban landscape VR imaging edge contour feature is solved as follows.


$$\begin{array}{c} S(s)=X_{0}exp\left(-\sum {\mathrm{\backslash nolimits}}_{x=0}^{t}\;\tau {\left(x^{*}\Delta i\right)}^{*}\Delta i\right) \end{array}$$ (14)

Where $\;\tau \left(x^{*}\Delta i\right)=w_{x}\rho$, where $w_{x}$ is the light intensity attenuation coefficient of the laser 3D scanned image of the urban landscape. The width of the imaging area $M=j_{max}-j_{min}$ and the height $B=k_{max}-k_{min}$, to obtain the 3D volume of the 3D laser scanning imaging area of the urban landscape $Q_{\text{box}\;}=L^{*}M^{*}B$. Let *N*1 be the absorption and scattering contrast of the 3D laser-scanned image of the urban landscape, and use the statistical feature analysis method to obtain the urban body drawing focal error ε of the VR design of the landscape is


$$\varepsilon =\frac{\lambda_{\chi }\sigma }{\sqrt{N}}$$ (15)

From the above, it can be seen that the urban landscape VR imaging body drawing is affected by the color component, which leads to imaging error. This study integrates two techniques: adaptive feature decomposition for point scanning and dynamic recognition of urban landscape images.A texture mapping method is then applied to systematically traverse the laser 3D-scanned image rays of the urban landscape in a layer-by-layer manner. The edge contour N(w, t) of the closed laser 3D scanned urban landscape imaging is constructed according to the KoKsma-Hlawka inequality [30]. The pixel enhancement value of the output virtual image is as follows.


$$\begin{array}{c} s_{PPM}(n)=\sum {\mathrm{\backslash nolimits}}_{x=-\infty }^{\infty }\;\sum {\mathrm{\backslash nolimits}}_{y=0}^{T_{u}-1}\;u\left(n-xN_{s}-yN_{u}-c_{y}N_{c}-g_{x}\varepsilon \right) \end{array}$$ (16)

The center pixel point localization is based on laser 3D scanning imaging technology to improve the dynamic feature point localization detection of the urban landscape. When the light projection center point σ≠0 of the VR design thread grid of the urban landscape, the spatial scanning of laser 3D scanning imaging is carried out along the fixed direction, thus realizing the texture segmentation of the urban landscape image.

### 3.2 Design optimization for virtual realization of urban landscape

#### 3.2.1 Image virtual reconstruction and filtering.

The order of error in laser 3D scanning imaging light attenuation is $O\left(N^{-\frac{1}{2}}\right)$, and if we represent the coordinate value of laser 3D scanning sampling as {(kZ,gz}, we can derive the following inequality:


$$\begin{array}{c} \left|\frac{1}{N}\sum {\mathrm{\backslash nolimits}}_{x=1}^{N}\;a\left(i_{x}\right)-\int_{0}^{1}\;a(n)dn\right|⩽Q(a)D_{N}^{*} \end{array}$$ (17)

The laser ray body plotting of the urban landscape 3D laser scanning urban landscape VR design is conducted and the number of points within the model is tallied. The grid model’s volume in the context of VR design for urban landscape can be described as follows.


$$Q_{obj}=Q_{box}\times \frac{U_{\text{xt}}}{U_{all}}$$ (18)

Where $Q_{\text{obj}\;}$ represents the ultimate volume measurement of the 3D urban landscape VR design model; $U_{in}$ signifies the count of points contained within the model; $U_{\text{all}}$ refers to the tally of all points that constitute the urban landscape design body. The trajectory flow of data points is crucial for achieving high-precision output in the 3D VR urban landscape design.

For instance, when reconstructing a virtual model of a city square, Equation (18) is used to calculate the volume of the square’s 3D model. The volume is estimated by calculating the ratio of laser-scanned points within the square’s boundaries ($U_{in}$) to the total scanned points ($U_{\text{all}}$). This ratio-based approach enables evaluation of the geometric accuracy between the virtual reconstruction and real-world dimensions.


$$Data\_term(i,j,d(i,j))=-\frac{{\left[\tau_{d}p,\tilde{p}\right]}_{\varphi_{i_{0}}}}{∥\tau_{d}p{∥}_{\varphi_{i_{0}}}∥\tilde{p}{∥}_{\varphi_{i_{0}}}}$$ (19)

Where $\tau_{d}p=p(i-w(i,j)-d(i,j),j)$ and the image attribute feature w(i,j) denotes the spatial building distribution distance. $[\cdot ,\cdot {]}_{\varphi_{i_{0}}}$ signifies the measurement error concerning urban landscape design with its center at point $i_{0}$. $∥\cdot {∥}_{\varphi_{i_{0}}}$ denotes the standardized parametrization of VR reproduction virtual imaging centered at point $i_{0}$. The correlation smoothing component of the image pixel distribution is derived from the edge contour feature details within the virtual image of the urban landscape.


$$Smooth\_term(\nabla d)=\Theta \cdot a\left(|\nabla \tilde{p}{|}^{2}\right)|\nabla d{|}^{2}+(1-\Theta )\Psi \left(|\nabla d{|}^{2}\right)$$ (20)

Where Θ is the image balance parameter of the urban landscape scene data; *a* is the degradation function of the 3D laser scanning, whose mathematical expression is


$$a(i)=e^{\frac{-i^{2}}{2\sigma^{2}}}$$ (21)

The terahertz wave image segmentation technique based on the adversarial generative network is used for image virtual reconstruction and filtering processing, combined with the adaptive feature decomposition method for point scanning and dynamic recognition of urban landscape images. The template function for urban landscape image recognition is automatically generated in the Scale-Space.


$$\begin{array}{c} N\left(i_{x+w},j_{x+t}\right)=\left\{ \begin{array}{c} @l1\;If\;r\left(i_{x+w},j_{x+t}\right)>s\\ 0\;If\;r\left(i_{x+w},j_{x+t}\right)⩽s \end{array}\right.\; \end{array}$$ (22)

Assuming the laser image S(i,j) of the 3D VR design of the VR urban landscape, the second-order moments of the pixel points L(i,j,σ) of the image after the rotation transformation are represented as U(i,j,L(i,j,σ)). The image virtual reconstruction and filtering process output is as follows.


$$\begin{array}{c} Y(i,j,\sigma )=\left(\frac{\partial U}{\partial i}\;\frac{\partial U}{\partial j}\right)=\left(\begin{array}{c} @l@1\;0\;L_{i}(i,j,\sigma )\\ 0\;1\;L_{j}(i,j,\sigma ) \end{array}\right) \end{array}$$ (23)

#### 3.2.2 VR output of 3D laser scanning of urban landscape.

The texture mapping method is used to traverse the laser 3D scanned image ray of the urban landscape layer by layer to obtain the chunking matrix of the 3D VR design of the urban landscape.


$$\begin{array}{c} W=\left(\begin{array}{c} @l@\frac{\partial^{2}U\to }{\partial i^{2}}\;\frac{\partial^{2}U\vec{T}}{\partial i\partial j}\\ \frac{\partial^{2}U\to \vec{T}}{\partial i\partial j}\;\frac{\partial^{2}U\to \vec{T}}{\partial j^{2}} \end{array}\right)=\left(\begin{array}{c} @l@\left(0,0,L_{ii}(i,j,\sigma )\right)\cdot \vec{T}\;\left(0,0,L_{ij}(i,j,\sigma )\right)\cdot \vec{T}\\ \left(0,0,L_{ij}(i,j,\sigma )\cdot \vec{T}\right)\;\left(0,0,L_{jj}(i,j,\sigma )\right)\cdot \vec{T} \end{array}\right)=\left(\begin{array}{c} @l@L_{ii}(i,j,\sigma )\;L_{ij}(i,j,\sigma )\\ L_{ij}(i,j,\sigma )\;L_{jj}(i,j,\sigma ) \end{array}\right) \end{array}$$ (24)

Calculate the frame recombination smoothing term for the 3D laser scanning and use the derivative method to obtain the central moments.


$$\frac{d}{di}F_{d_{i}}+\frac{d}{dj}F_{d_{j}}=2\left(\begin{array}{c} @l@\Theta \cdot div\left(a\left(|\nabla \tilde{p}{|}^{2}\right)\nabla d\right)+\\ (1-\Theta )\cdot div\left(\Psi^{r}\left(|\nabla d{|}^{2}\right)\nabla d\right) \end{array}\right)$$ (25)

Where the expression of the traversal function ψ’ for laser 3D scanning is given by


$$\Psi^{\prime }\left(s^{2}\right)=\frac{1}{\sqrt{1+\frac{s^{2}}{\beta^{2}}}}$$ (26)

Combining the adaptive feature decomposition method for point scanning and dynamic recognition of urban landscape images, the virtual reconstruction model of urban landscape design is obtained as follows.


$$D_{x}-\alpha \left(\begin{array}{c} @l@\Theta_{x}\sum {\mathrm{\backslash nolimits}}_{y\in T(x)}\;\frac{a_{x}+a_{y}}{2}\left(d_{y}-d_{x}\right)+\\ \left(1-\Theta_{x}\right)\sum {\mathrm{\backslash nolimits}}_{y\in T(x)}\;\frac{\Psi_{x}+\Psi_{y}}{2}\left(d_{y}-d_{x}\right) \end{array}\right)=0$$ (27)

The images of VR output are rendered into frames to extract the information feature values of VR reproduction virtual imaging of urban landscape to realize the VR design of urban landscape.


$$W=\left[\begin{array}{c} @l@p_{11}\;p_{12}\\ p_{21}\;p_{22} \end{array}\right]=p\left(i,j,\sigma_{X},\sigma_{D}\right)=\sigma_{D}^{2}A\left(\sigma_{X}\right)*\left[\begin{array}{c} @l@L_{i}^{2}\left(i,j,\sigma_{D}\right)\;L_{i}L_{j}\left(i,j,\sigma_{D}\right)\\ L_{i}L_{j}\left(i,j,\sigma_{D}\right)\;L_{j}^{2}\left(i,j,\sigma_{D}\right) \end{array}\right]$$ (28)

**Fig 3:**
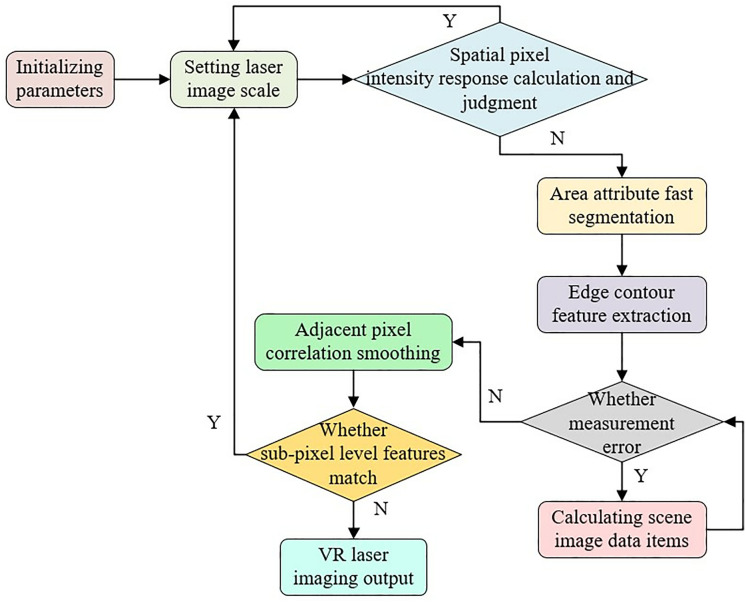
Flow chart of the proposed design technology implementation.

Where $A\left(\sigma_{\text{I}}\right)$ denotes the neighborhood of the urban landscape VR design; $\sigma_{X}$is the edge contour scale of each pixel point; $\sigma_{D}$ is the differential scale; *i, j* are the neighborhood points of the landscape design. $L\left(i,j,\sigma_{\text{D}}\right)$ denotes the traces of the VR virtual image on each scale $\sigma^{\left(t\right)}(1,2,\cdots ,t)$. $L_{i}(i,j,\;\sigma_{D})$, $L_{j}\left(i,j,\sigma_{D}\right)$ denote the initial image scale and the scale of the urban landscape after virtual reconstruction, respectively. In summary, the algorithm implementation flow designed in this paper is obtained as shown in Fig 3.

The training time of our GAN model depends on the dataset size and network complexity. For a dataset of N images with resolution *H* × *W*, the training time scales as O(*N* × *H* × *W* × *E*), where E is the number of epochs. In our experiments, with *N* = 1000, *H* = 256, *W* = 256, and *E* = 50, training took approximately 12 hours on an NVIDIA RTX 2070 GPU. Inference time, critical for real-time applications, averages 0.5 seconds per image, leveraging GPU parallelization and an optimized network design. Compared to traditional tools like 3D Studio Max, which may require minutes per frame, our method offers significant speed improvements.

Memory usage is driven by model parameters and intermediate computations. Our GAN, with a generator of 10 residual blocks and a discriminator of 5 convolutional layers, has approximately 25 million parameters, requiring about 100 MB of storage. Training peaks at 8 GB of memory, including input batches and gradients, while inference uses around 2 GB, suitable for standard hardware. Techniques like gradient checkpointing and mixed-precision training help optimize memory consumption.

We benchmarked our method against tools like SketchUp and 3D Studio Max. For a scene with 479 objects and 743,007 planes, our method rendered in 49.6 seconds, far faster than traditional tools. Compared to StyleGAN (over 100 million parameters), our model’s 25 million parameters highlight its efficiency.

## 4. Result analysis and discussion

### 4.1 Experimental setup

The design technology performance verification simulation process, set up the test hardware accessories platform is as follows: the system uses Windows 10 Professional Edition version 1809, the platform for the processor Rui Long R7 2700, 8 cores and 16 threads frequency locked to 4.0GHZ, memory is Chic Phantom Halberd 16G DDR4 3000, graphics card is ShadowChip RTX2070.

### 4.2 VR simulation scene experiment and analysis

This experiment designed a VR simulation scene of a small urban architectural landscape. SketchUp, 3D Studio Max and the design technology of this paper are used to analyze the difference and superiority through the comparison of data such as rendering speed, image processing performance and image quality. In order to highlight the accuracy of the proposed virtual scene design, the simulated simulation rendering experiments of the scene were conducted in four times based on four aspects: number of objects, number of planes, number of object sides, and decorative vegetation/trees in four environments: simple scenario, complex scenario, 2 times complex scenario, and 3x complex scenario (as shown in Table 1), on the same experimental platform infrastructure equipment. It was tested that there were great differences between different software design methods in terms of the number of model libraries, operation difficulty, rendering time, and difficulty of output (see Table 2). These differences also have an important impact on the effect of the final scene.

**Table 1:** Rendering environment parameters for scenes of different complexity.

No.	Name of the test scenario	Simple scenario	Complex scenario	2 times complex scenario	3 times complex scenario
1	Number of objects	82	167	323	479
2	Number of planes	22459	247743	495375	743007
3	Number of object sides	103515	1445155	2890199	43243
4	Decorative vegetation/trees	10	80	150	250
5	Roofs, tiles, window sculptures, etc.	5	18	25	46

**Table 2:** Performance differences between different simulation design methods.

Method	Difficulty of modeling operation	Rendering operation difficulty	Rendering animation function	Model libraries that can be called directly	Real-time rendering function
SketchUp	Moderate	Complicated	Moderate	No	No
3D Studio Max	Complex	Complicated	Complex	No	No
Proposed	Simple	Simple	Simple	Yes	Yes

#### 4.2.1 Analysis of the variability in time spent.

**Fig 4:**
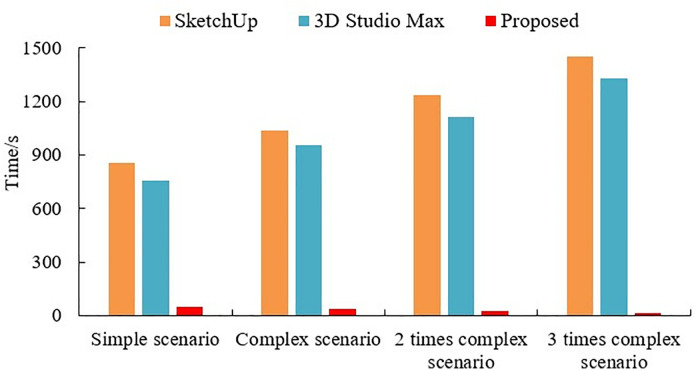
Comparison of initial rendering time across scenarios of increasing complexity.

**Fig 5:**
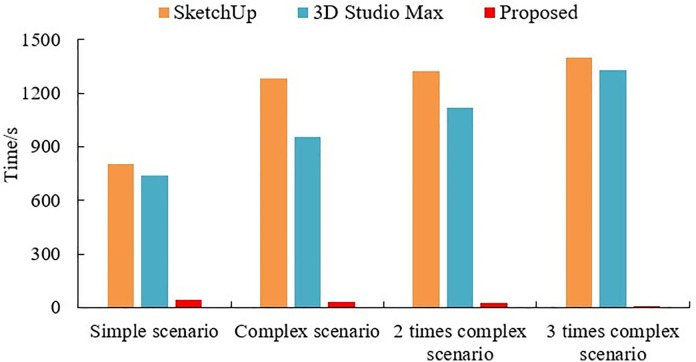
Comparison of final rendering time and scalability across multi-scene batches.

The frequency of using batch rendering objects in the simulation landscape design process is high, so speed is also a key factor affecting efficiency. The time consumed by the proposed design method is between 10.8–49.6 seconds, which saves 691.7–1318.9 seconds compared to the rendering method used by 3D Max in the same scene; Compared to SketchUp’s rendering method, it saves 755.8–1441.5 seconds (see Figs 4 and 5). The experimental data results show that the method designed in this paper has the advantage of faster efficiency. Because this paper uses 3D laser 3D scanning imaging technology for VR imaging rendering body drawing will greatly improve the efficiency of scene file processing. Moreover, as the complexity of the scene increases, the rendering time is smooth and evenly distributed, much lower than the other two methods.

#### 4.2.2 Analysis of effect variability.

**Fig 6:**
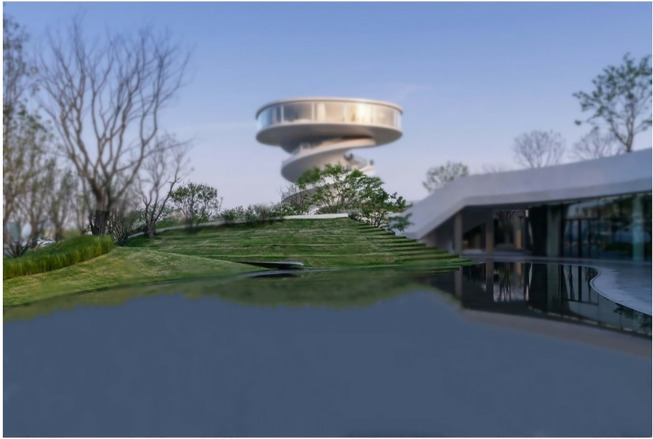
SketchUp experimental test result.

**Fig 7:**
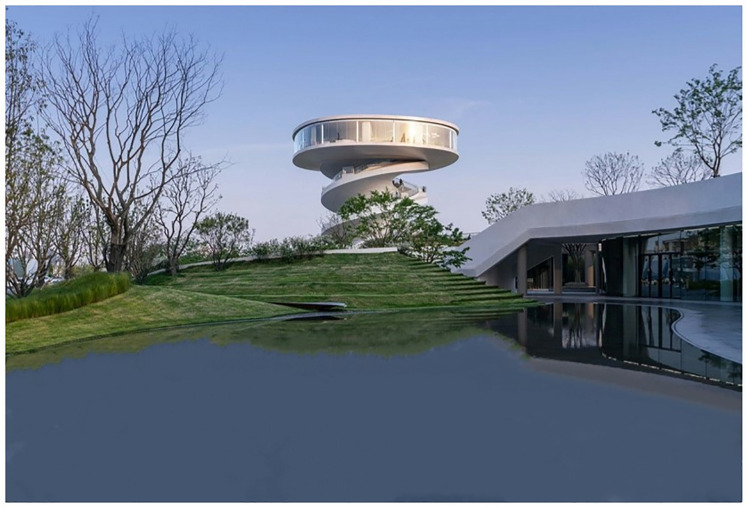
3D Studio Max experimental test result.

**Fig 8:**
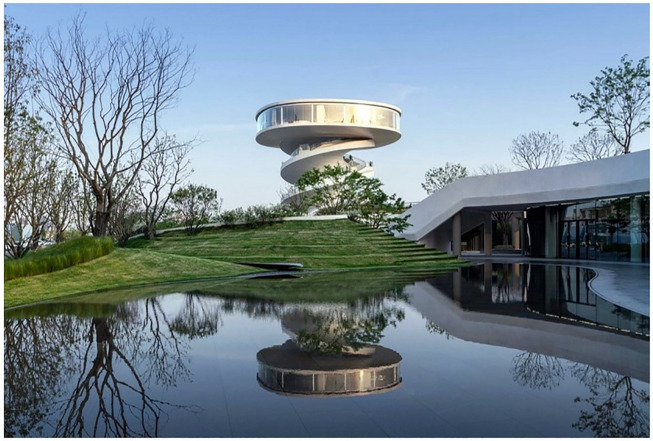
Result of the proposed method.

As shown in Table 3, the proposed method achieves rapid rendering while significantly enhancing scene lighting effects (reflection/refraction), architectural realism, and landscape detail precision. These improvements demonstrate superior output quality compared to traditional approaches. The simulation technique designed in this paper produces real-time results and can save the image generated by the graphics card as an image file or animation. The simulation effect produced by 3D Studio Max is slightly inferior, and it relies on Adobe Photoshop to produce effects such as landscape plants and river details, which brings complexity to the work. SketchUp’s effect is more inferior to the first two in terms of spatial color performance, the overall picture is blurred and lacks realism, and the materials are single (see Figs 6–8).

**Table 3:** Analysis of the differences in the effects of rendering complex scenes.

Method	Clarity of rendering	Indoor light and shadow effect	Outdoor light and shadow effect	Naturalness of model effect	Naturalness of background	Model fineness
SketchUp	High	Mid-high	Low	Low	Low	Low
3D Studio Max	High	High	Middle	Middle	Low	Mid-high
Proposed	High	High	High	High	High	High

To enhance the objectivity of our evaluation, we have incorporated quantitative metrics, including Peak Signal-to-Noise Ratio (PSNR), Structural Similarity Index (SSIM), and Learned Perceptual Image Patch Similarity (LPIPS). PSNR and SSIM were computed for cases with reference images (e.g., ground truth from 3D laser scans), while LPIPS was used to assess perceptual realism in the absence of references. These metrics were applied to the rendered outputs of our method, SketchUp, and 3D Studio Max. As shown in Table 4, our method achieves higher PSNR (33.7 dB) and SSIM (0.92) values, indicating superior image fidelity, and a lower LPIPS score (0.12), suggesting better perceptual quality.

**Table 4:** Quantitative comparison of image quality metrics.

Method	PSNR (dB)	SSIM	LPIPS
SketchUp	28.5	0.85	0.25
3D Studio Max	30.2	0.88	0.18
Proposed	33.7	0.92	0.12

To further substantiate these findings, we performed a one-way ANOVA on the PSNR, SSIM, and LPIPS values across the three methods (see Table 5). The results indicate significant differences between the methods for all three metrics (p < 0.01). Post-hoc Tukey tests reveal that our method significantly outperforms both SketchUp and 3D Studio Max in terms of PSNR (p < 0.05), SSIM (p < 0.05), and LPIPS (p < 0.01), with the most pronounced improvement in perceptual quality as measured by LPIPS. These statistical outcomes reinforce the qualitative superiority of our method and provide a robust basis for its advantages in visual fidelity.

**Table 5:** Summary of ANOVA and Post-hoc Tukey test results.

Metric	ANOVA p-value	Proposed vs. SketchUp (Tukey p)	Proposed vs. 3D Studio Max (Tukey p)
PSNR	<0.01	<0.05	<0.05
SSIM	<0.01	<0.05	<0.05
LPIPS	<0.01	<0.01	<0.01

## 5. Conclusion

In the contemporary milieu of urban development, the exploration of optimum methodologies for urban landscape design is pivotal in fostering progressive construction initiatives. Within this context, the present research delineates a groundbreaking approach to urban landscape design that leverages frame reorganization and texture mapping facilitated through 3D laser scanning technologies. Central to this approach is the realization of an exhaustive three-dimensional simulation of urban architectural landscapes, which demands the transformative reorganization of their innate three-dimensional frameworks. This is actualized through a meticulous procedure involving the acquisition of central pixel points of imagery via laser scanning. Following this, the gathered data undergoes a sophisticated process of reorganization and filtration utilizing terahertz wave image segmentation technology, which operates on the principles of adversarial generative networks. Building upon this foundation, an adaptive decomposition methodology is engaged to undertake the pixel point scanning regimen of the urban imagery, thereby facilitating dynamic image discernment and the subsequent acquisition of dynamic imagery. This intricate process holds the potential to substantially augment the perceptual depth and texture of urban landscapes in simulation environments. In substantiating the proposed methodology’s efficacy and superior design outcomes, simulation experiments were conducted. The findings elucidate the remarkable efficiency and design potency availed by this innovative method in the context of urban landscape design, heralding a promising avenue for future urban development projects.

However, it is acknowledged that the current experimental setup primarily evaluates the method under controlled conditions and does not fully account for real-world noise factors such as weather conditions, seasonal variations, and pedestrian movement, which are critical for practical urban landscape design applications. To address this limitation and enhance the method’s robustness, future work will include the following enhancements: (1) collecting diverse datasets with images captured under various weather conditions (e.g., sunny, rainy, foggy), across different seasons (e.g., spring, summer, autumn, winter), and with varying pedestrian activity levels (e.g., low, medium, high foot traffic). (2) implementing data augmentation techniques, such as altering lighting or adding synthetic occlusions, to simulate environmental variability.

## References

[1] Xu Y, Liu M, Hu Y, Li C, Xiong Z. Analysis of Three-Dimensional Space Expansion Characteristics in Old Industrial Area Renewal Using GIS and Barista: A Case Study of Tiexi District, Shenyang, China. Sustainability. 2019;11(7):1860. doi: 10.3390/su11071860

[2] Borkowski A, Nowakowski P. Use of applications and rendering engines in architectural design – state-of-the-art. Bud-Arch. 2023;22(1):005–14. doi: 10.35784/bud-arch.3327

[3] Hajirasouli A, Banihashemi S, Sanders P, Rahimian F. BIM-enabled virtual reality (VR)-based pedagogical framework in architectural design studios. SASBE. 2023;13(6):1490–510. doi: 10.1108/sasbe-07-2022-0149

[4] Matthews B, Shannon B, Roxburgh M. Destroy All Humans: The Dematerialisation of the Designer in an Age of Automation and its Impact on Graphic Design—A Literature Review. Int J Art Design Ed. 2023;42(3):367–83. doi: 10.1111/jade.12460

[5] Zhou M, Wang J, Yu B, Chen K. A Quality Management Method for Prefabricated Building Design Based on BIM and VR-Integrated Technology. Applied Sciences. 2024;14(4):1635. doi: 10.3390/app14041635

[6] Souza MP de, Fabricio MM. BIM and virtual reality for pre-design evaluation of building performance. Architectural Engineering and Design Management. 2024;20(5):1260–79. doi: 10.1080/17452007.2024.2388714

[7] Yan C. Utilizing Digital Art Virtual Reconstruction Technology in the Construction Industry for Modern Urban Landscape Sculpture Planning and Design. Computer-Aided Design & Applications. 2023;55–69. doi: 10.14733/cadaps.2024.s11.55-69

[8] Lin Z, Wang Y, Ye X, Wan Y, Lu T, Han Y. Effects of Low-Carbon Visualizations in Landscape Design Based on Virtual Eye-Movement Behavior Preference. Land. 2022;11(6):782. doi: 10.3390/land11060782

[9] Yu Z, Si Z, Li X, Wang D, Song H. A Novel Hybrid Particle Swarm Optimization Algorithm for Path Planning of UAVs. IEEE Internet Things J. 2022;9(22):22547–58. doi: 10.1109/jiot.2022.3182798

[10] Do TLP, Coffin M, Gentet P, Hwang L, Lee S. Development of a Tabletop Hologram for Spatial Visualization: Application in the Field of Architectural and Urban Design. Buildings. 2024;14(7):2030. doi: 10.3390/buildings14072030

[11] Janovský M. Pre-Dam Vltava River Valley—A Case Study of 3D Visualization of Large-Scale GIS Datasets in Unreal Engine. IJGI. 2024;13(10):344. doi: 10.3390/ijgi13100344

[12] Cook M, Lischer-Katz Z, Hall N, Hardesty J, Johnson J, McDonald R, et al. Challenges and Strategies for Educational Virtual Reality. ITAL. 2019;38(4):25–48. doi: 10.6017/ital.v38i4.11075

[13] Chen J, Wang W, Fang B, Liu Y, Yu K, Leung VCM, et al. Digital Twin Empowered Wireless Healthcare Monitoring for Smart Home. IEEE J Select Areas Commun. 2023;41(11):3662–76. doi: 10.1109/jsac.2023.3310097

[14] Tadeja SK, Seshadri P, Kristensson PO. AeroVR: An immersive visualisation system for aerospace design and digital twinning in virtual reality. Aeronaut J. 2020;124(1280):1615–35. doi: 10.1017/aer.2020.49

[15] Javaid M, Haleem A. Virtual reality applications toward medical field. Clinical Epidemiology and Global Health. 2020;8(2):600–5. doi: 10.1016/j.cegh.2019.12.010

[16] Blinder D., Birnbaum T., Ito T., Shimobaba T. (2022). The state-of-the-art in computer generated holography for 3D display. Light: Advanced Manufacturing, 3(3), 572–600.

[17] Bashabsheh AK, Alzoubi HH, Ali MZ. The application of virtual reality technology in architectural pedagogy for building constructions. Alexandria Engineering J. 2019;58(2):713–23. doi: 10.1016/j.aej.2019.06.002

[18] Marks B, Thomas J. Adoption of virtual reality technology in higher education: An evaluation of five teaching semesters in a purpose-designed laboratory. Educ Inf Technol (Dordr). 2022;27(1):1287–305. doi: 10.1007/s10639-021-10653-6 34257511 PMC8265284

[19] Mertz L. Virtual Reality Pioneer Tom Furness on the Past, Present, and Future of VR in Health Care. IEEE Pulse. 2019;10(3):9–11. doi: 10.1109/MPULS.2019.2911808 31135344

[20] Ketzler B, Naserentin V, Latino F, Zangelidis C, Thuvander L, Logg A. Digital Twins for Cities: A State of the Art Review. built environ. 2020;46(4):547–73. doi: 10.2148/benv.46.4.547

[21] Sadhu A, Peplinski JE, Mohammadkhorasani A, Moreu F. A Review of Data Management and Visualization Techniques for Structural Health Monitoring Using BIM and Virtual or Augmented Reality. J Struct Eng. 2023;149(1). doi: 10.1061/(asce)st.1943-541x.0003498

[22] Dembski F, Wössner U, Letzgus M, Ruddat M, Yamu C. Urban Digital Twins for Smart Cities and Citizens: The Case Study of Herrenberg, Germany. Sustainability. 2020;12(6):2307. doi: 10.3390/su12062307

[23] Akpan IJ, Shanker M. A comparative evaluation of the effectiveness of virtual reality, 3D visualization and 2D visual interactive simulation: an exploratory meta-analysis. SIMULATION. 2018;95(2):145–70. doi: 10.1177/0037549718757039

[24] Tseng P-H, Hung S-H, Chiang P-Y, Yao C-Y, Chu H-K. EZ-Manipulator: Designing a mobile, fast, and ambiguity-free 3D manipulation interface using smartphones. Comp Visual Media. 2018;4(2):139–47. doi: 10.1007/s41095-018-0105-0

[25] Voci P. Para-animation in Practice and Theory: The Animateur, the Embodied Gesture and Enchantment. Animation. 2023;18(1):23–41. doi: 10.1177/17468477231155543

[26] Wang X, Zhen F, Tang J, Shen L, Liu D. Applications, Experiences, and Challenges of Smart Tourism Development in China. J Urban Technology. 2021;29(4):101–26. doi: 10.1080/10630732.2021.1879605

[27] Liu X, Zheng W, Mou Y, Li Y, Yin L. Microscopic 3D reconstruction based on point cloud data generated using defocused images. Measurement and Control. 2021;54(9–10):1309–18. doi: 10.1177/00202940211033881

[28] Zhu W, Dong F, Hou B, Kenniard Takudzwa Gwatidzo W, Zhou L, Li G. Segmenting the Semi-Conductive Shielding Layer of Cable Slice Images Using the Convolutional Neural Network. Polymers (Basel). 2020;12(9):2085. doi: 10.3390/polym12092085 32937761 PMC7569897

[29] Maqsood MH, Mumtaz R, Haq IU, Shafi U, Zaidi SMH, Hafeez M. Super Resolution Generative Adversarial Network (SRGANs) for Wheat Stripe Rust Classification. Sensors (Basel). 2021;21(23):7903. doi: 10.3390/s21237903 34883905 PMC8659936

[30] Ermakov S, Leora S. Monte Carlo Methods and the Koksma-Hlawka Inequality. Mathematics. 2019;7(8):725. doi: 10.3390/math7080725

